# Adipose-derived mesenchymal stem cells and platelet-rich plasma synergistically ameliorate the surgical-induced osteoarthritis in Beagle dogs

**DOI:** 10.1186/s13018-016-0342-9

**Published:** 2016-01-15

**Authors:** Sungho Yun, Sae-Kwang Ku, Young-Sam Kwon

**Affiliations:** Department of Veterinary Surgery, College of Veterinary Medicine, Kyungpook National University, Daegu, 702-701 Republic of Korea; Department of Anatomy and Histology, College of Korean Medicine, Daegu Haany University, Gyeongsan, 712-715 Republic of Korea; Stem Cell Therapeutic Research Institute, Kyungpook National University, Daegu, 702-701 Republic of Korea

**Keywords:** Adipose-derived MSC, Canine, Osteoarthritis, Platelet-rich plasma

## Abstract

**Background:**

The purpose of this study is to investigate the clinical effects of platelet-rich plasma (PRP) and adipose-derived mesenchymal stem cell (MSC) as the fundamental treatment of osteoarthritis (OA).

**Methods:**

Twenty four Beagle dogs were used as cranial cruciate ligament transection models. The dogs were divided into four groups (*n* = 6) according to the intra-articular injection materials: the control group with phosphate-buffered saline (PBS), the PRP group with PRP, the MSC group with MSCs emerged in PBS, and the MSC and PRP co-treatment (MP) group with MSCs and PRP.

**Results:**

Lameness score, focal compression strength, articular extracellular matrix (ECM) compositions, histopathology, and real-time PCR were used to evaluate the effects of PRP and MSCs on canine OA. In the order of MP, PRP, and MSC group, these all showed positive effects on the evaluated categories. The lameness scores were lower, and the focal compression strengths of the affected femoral articular surface cartilages were higher than those in the OA control group. Also, the inflammatory changes, when evaluated with Mankin scoring and histomorphologic examination, were significantly ameliorated with the treatment of PRP and/or MSCs. The glycosaminoglycan and collagen composition of extracellular matrix was more favorable in the test groups. The ECM-related genes significantly increased through the up-regulation, while the protein expressions of inflammatory cytokines were decreased through the inhibitory effects of PRP and MSCs on chondrocyte apoptosis and inflammatory cytokines.

**Conclusions:**

Taken together, this study suggests that PRP and MSCs treatments have a beneficial effect on OA via the stimulation of ECM synthesis and chondrocyte proliferation and via the inhibition of inflammatory reaction.

**Electronic supplementary material:**

The online version of this article (doi:10.1186/s13018-016-0342-9) contains supplementary material, which is available to authorized users.

## Background

Osteoarthritis (OA) is the most common clinical syndrome of joint pain and dysfunction, accompanied by varying degrees of functional limitation and the reduced quality of life. Because it is mostly irreversible and progressive, OA joint consequently loses their cartilage layer [1].

The most ideal treatment of OA is focused on blocking the catabolic activity of cartilage and enhancing regeneration of normal cartilage. Until now, therapies for the OA commonly focused on the palliation of pain and discomfort, improvement on functional movement, and prevention of further degeneration. Thus, the primary approach in the clinical treatment of OA involves the extensive use of NSAIDs, analgesics, and hyaluronan which allows the brief symptomatic relief but provides no apparent disease-modifying effect [2–4]. Therefore, there is a critical need for the development of the alternative agents that can fundamentally prevent the destruction of cartilages or stimulate its proper repair. In these aspects, various efforts have been tried to search for the effective cartilage-preserving methods with cell sources.

To sum up, the isolated chondrocyte expansion and implantation method is regarded as the fundamental solution. However, the main concern of the most cultured chondrocytes is losing the characteristics of producing hyaline-like cartilage [5, 6]. In a canine model, it has been reported that cultured autologous chondrocytes failed to return to the normal hyaline cartilage [7].

In human, the intra-articular injection of 1.0 × 10^8^ cells of adipose-derived mesenchymal stem cell (MSC) could improve the function, reduce the pain, and regenerate the hyaline-like cartilage [8]. MSCs are typically not observed in synovial fluid. However, MSCs appear in synovial fluid in the OA condition, and they are thought to play an important role in the regeneration of damaged tissue and have anti-inflammatory effect [9]. As the patient ages, the quality and quantity of MSCs decrease and there is a reduction of their ability to proliferate and differentiate. Also, the depletion of healthy MSCs is regarded as one of the reasons worsening OA [10]. For these reasons, it is believed that the direct administration of MSCs could promote the positive role of them by preventing their depletion and improving cartilage regeneration [11].

The platelet-rich plasma (PRP) is defined as the plasma with the platelet population of more than 1.0 × 10^6^ cells/μl, and it has typically four- to eightfold more platelets than the normal plasma [12]. PRP has various growth factors such as platelet-derived growth factor and transforming growth factor beta [13]. It has been well known that PRP has angiogenic, anti-inflammatory, and anti-catabolic effects [13, 14]. It has also been reported that transforming growth factor beta and fibroblast growth factor from PRP have an anabolic effect on cartilage metabolism [15]. These factors not only regulate the cell migration and proliferation but also enhance the wound healing and extracellular matrix (ECM) remodeling via the stimulation of angiogenesis [16, 17].

Based on the previous studies, it is hypothesized that the PRP could have a synergistic effect on the cartilage regeneration with the combination of MSCs. Therefore, the purpose of this study is to examine the effect of PRP and MSCs on the morphologic change and regeneration of articular cartilage in the inflammation process using canine OA model.

## Methods

### PRP preparation

Autologous PRP was prepared in each dog using double spin method. Fifty milliliters of fresh blood was collected with 7 ml of acid citrate dextrose formula A. Then, the blood was centrifuged at 1200 rpm for 10 min into three layers: plasma, buffy coat, and red blood cell. After the plasma and buffy coat were separated into a new tube, the mixture was centrifuged at 2500 rpm for 10 min. Discarding the supernatant, only the lower 20 % of the plasma was harvested. The collected plasma (PRP) was tested using complete blood cell count test (Cell-Dyne, Abbott Lab., USA) to make sure it had 1.0 × 10^6^ platelets/μl or more. All prepared PRP in this study were used within 6 h.

### Adipose-derived mesenchymal stem cell isolation and culture

Approximately 15 g of fat tissue was aseptically collected from the flank of a dog. It was rinsed with phosphate-buffered saline (PBS) several times to remove any remaining anesthetic agent and blood. Rinsed fat tissue was then digested using 0.075 % collagenase type I (collagenase type 1A, Sigma-Aldrich, USA) in 37 °C water-chamber for 2 h with shaking or inverting every 30 min. After adding the equal volumes of Dulbecco modified Eagle medium and 10 % fetal bovine serum, it was centrifuged at 1200 rpm for 10 min. The supernatant and digested lipids were discarded. The cell pellet was washed with PBS, and it was filtered through 100-μm nylon mesh. After centrifuging at the same condition, the cells were suspended into a 100 × 20 mm cell culture dish with low-glucose Dulbecco modified Eagle medium and 10 % fetal bovine serum. After 24 h, non-adherent cells and debris were washed with PBS, and cell culture media was replaced twice per week. MSCs were collected and used between passage 1 and 2 in all the experiments of this paper. The flow cytometry analysis with established MSCs was performed, and obtained MSCs were negative for cluster of differentiation (CD) 34 and CD45 and strongly positive for CD29 and CD44 (Additional file 1: Fig. S6).

### The canine model of cranial cruciate ligament transection

The procedures were approved by Institutional Animal Care and Use Committees of Kyungpook National University. Twenty four physically healthy Beagle dogs were used in this experiment. The weights of dogs were 7.7 ± 1.1 kg (mean ± standard deviation), and ages were between 2–3 years old. Under the anesthetic state, the cranial cruciate ligament of a right hind limb was excised with a no. 11 scalpel blade. The connective tissues and skin were sutured with routine procedure. Analgesics (tramadol 8 mg/kg BID, subcutaneous) and antibiotics (enrofloxacin 5 mg/kg SID, subcutaneous) were administrated for 3 days after the surgery. After a week for soft tissue healing, each dog regularly walked for 10 min per day for 2 months. Then, treatment was given every week for 1 month. Another 2 months later, the dogs were sacrificed and stifle samples were collected.

### MSC and PRP application

After the canine OA model, the subjects were treated every week for 1 month with an intra-articular injection with each material according to the groups: the control group with 1 ml of PBS, the PRP group with 1 ml of PRP, the MSC group with 1.0 × 10^7^ MSCs in 1 ml of PBS, and the MSC and PRP co-treatment (MP) group with 1.0 × 10^7^ MSCs in 1 ml of PRP. The contralateral stifle joint of the dogs in control group were used as a sham group in a histopathological examination, and no treatment material was given.

### Evaluations

#### Lameness score

The lameness score was measured before the surgery and by every month after the surgery. All dogs had normal gait and no lameness before surgery. Previously described scoring system was used [18], and is as followed: 0, no detectable lameness; 1, minor lateral weight shift but no lameness at walk and trot; 2, no lameness at a walk but mild lameness at a trot; 3, mild lameness at a walk and significant lameness at trot; 4, non-weight bearing at a trot; and 5, non-weight bearing at walk and standing. As a blind test, three veterinarians assessed the grade of lameness.

#### The measurement of focal compressive strengths

After sacrifice and sampling, the ex vivo compression strengths of the femoral and tibial articular surfaces (0.2 mm) of each sample were detected with a computerized testing machine (SV-H1000, Japan Instrumentation System Co., Tokyo, Japan) as *N* (Newton). The measured points were central region of the medial femoral and tibial condyle.

#### Histological process

The articular cartilages of central region of the lateral femoral and tibial condyle were taken from knee joints of each group, and they were separately fixed in 10 % neutral buffered formalin (NBF), and then decalcified in decalcifying solution (24.4 % formic acid and 0.5 N sodium hydroxide) for 14 days (mixed decalcifying solution was exchanged once a day for 14 days). Each femoral and tibial articular surface cartilage was longitudinally trimmed, then embedded in paraffin, sectioned (3–4 μm) using tungsten bladder equipped automated polycut microtome (Model RM2255, Leica, Wetzlar, Germany), and stained with Sirius red stain for cartilaginous tissues. In each prepared histological samples, the histological profiles were interpreted under a light microscope (Model Eclipse 80*i*, Nikkon, Tokyo, Japan) as blinds to group distribution when this analysis was made.

#### Analysis of ECM compositions

Some parts of the cartilage on the femoral and tibial articular surface taken from the knee joints of each group were separately lyophilized to obtain the dry weight. Then, the piece was digested and used for glycosaminoglycan (GAG) and collagen (COL) analyses [19, 20]. The concentration of GAG was determined through the di-methyl-methylene blue sulfated GAG assay using a UV/VIS spectrophotometer (Optizen Pop, Mecasys, Daejeon, Korea). Collagen content was determined by Erlich’s hydroxyproline assay [21]. The hydroxyproline content was converted to the collagen content using the following equation: (μg hydroxyproline × dilution factor)/0.13 = μg collagen, based on the fact that hydroxyproline representing approximately 13 % of the amino acid content of collagen in the human meniscus [22]. The concentrations of GAG and collagen were standardized to tissue dry weight and expressed as microgram per milligram to allow comparison among the experimental groups.

#### ECM-related chondrogenic gene mRNA expressions

The SOX9 and aggrecan messenger RNA (mRNA) expressions on the femoral and tibial articular surface cartilages were detected using real-time PCR. Briefly, RNA was extracted using Trizol reagent (Invitrogen, Carlsbad, CA, USA). The RNA concentrations and quality were determined by CFX96™ Real-Time System (Bio-Rad, Hercules, CA, USA). To remove contaminating DNA, samples were treated with recombinant DNase I (DNA-free; Ambion, Austin, TX, USA). RNA was reverse transcribed using the reagent High-Capacity cDNA Reverse Transcription Kit (Applied Biosystems, Foster City, CA, USA) according to the manufacturer’s instructions. The cDNA strand was synthesized from the total RNA and then the mixture of primers and the cDNA products was amplified by PCR, and the conditions of PCR amplification were 58 °C for 30 min, 94 °C for 2 min, 35 cycles of 94 °C for 15 s, 60 °C for 30 s, 68 °C for 1 min, and then 72 °C for 5 min. Analysis was carried out using ABI Step One Plus Sequence Detection System (Applied Biosystems, Foster City, CA, USA), and their expression levels were calculated as relative to sham group. The expression of glyceraldehyde 3-phosphate dehydrogenase (G3PDH) mRNA was used as a control for tissue integrity in all of the samples. The sequences of the PCR oligonucleotide primers were as listed in Table 1.

**Table 1:** Oligonucleotides for real-time PCR used in this study

Target	5′–3′	Sequence	NCBI accession no.
SOX9	Sense	AAGCTCTGGAGGCTGCTGAA	NM_001002978.1
	Antisense	ACTTGTAATCCGGGTGGTCTTTC	
Aggrecan	Sense	CTATGAGGACGGCTTTCACC	U65989.2
	Antisense	AGACCTCACCCTCCATCTCC	
G3PDH	Sense	TATTGTCGCCATCAATGACC	NM_01003142
	Antisense	TACTCAGCACCAGCATCACC	

*PCR* polymerase chain reaction, *NCBI* National Center for Biotechnology Information

#### BrdU uptake measurement

To assess the effects of MSC and PRP or their co-treatment (MP) on the proliferation of cells within the dog knee joints, proliferating cells were labeled by an intraperitoneal injection of 5-bromo-2′-deoxyuridine (BrdU). Dogs were given intraperitoneal injections of BrdU (Sigma-Aldrich, St. Louise, MO, USA) 50 mg/kg, in a volume of 1 ml/kg and dissolved in saline, and the animals were sacrificed 72 h later. BrdU uptakes were detected with immunohistochemistry using an anti-BrdU antibody, as shown in histomorphometry sections.

#### Immunohistochemistry

Immunoreactivity for BrdU as cell proliferating marker was considered using purified primary antibody with avidin-biotin-peroxidase complex (ABC). Immunoreactivities in the prepared femoral and tibial surface cartilage tissues against caspase-3, cleaved poly(ADP-ribose) polymerase (PARP), tumor necrosis factor (TNF)-α, cyclooxygenase (COX)-2, interleukin (IL)-1β, interferon (IFN)-γ, and inducible nitric oxide synthase (iNOS) were also additionally observed after treatment MSCs, PRP, or their combination. Briefly, endogenous peroxidase activity was blocked by incubated in methanol and 0.3 % H_2_O_2_ for 30 min, and non-specific binding of immunoglobulin was blocked with normal horse serum blocking solution for 1 h in humidity chamber after epitope retrievals by pretreatment of trypsin (Sigma-Aldrich, St. Louise, MO, USA) and 2 N HCl, on the prepared unstained sections. The primary antisera were treated for overnight at 4 °C in humidity chamber and then incubated with biotinylated universal secondary antibody and ABC reagents for 1 h at room temperature in humidity chamber. Finally, sections were reacted with peroxidase substrate kit for 3 min at room temperature. All sections were rinse in 0.01 M PBS for three times, between each step. The primary antisera and detection kits for immunohistochemistry used in this study were described in Additional file 1: Table S1. 

#### Histomorphometry

To observe more detailed histopathological changes, the articular cartilage injuries stained with Safranin O staining were evaluated and recorded using the Mankin scoring systems referred by the other studies [23]. With this system, the higher the score, the higher the level of OA (semiquantative scores; max = 12). The thicknesses of femur and tibia articular cartilages (micrometer per cartilage) were measured with the histomorphometrical analyses at prepared longitudinally trimmed samples, using a computer based automated image analyzer (*i*Solution FL ver 9.1, IMT *i*-solution Inc., Vancouver, Quebec, Canada). A total of six histological regions of femoral and tibial articular surface regions of each group were considered for further analysis. The cells occupied by over 20 % of immunoreactivities, the density, of each antibody for caspase-3, PARP, TNF-α, COX-2, IL-1β, IFN-γ, and iNOS were regarded as positive, and the numbers of each immunoreactive cells were counted separately in each of the femoral and tibial articular surface regions as cells per square millimeter, under blinds condition.

### Statistical analysis

The values were expressed as mean ± standard deviation (SD). A multiple comparison tests were conducted for different groups. According to the result of variance homogeneity by the Levene test, one-way ANOVA test and the least-significant differences (LSD) multi-comparison test were used for parametric comparison, and Kruskal-Wallis H test was used for non-parametric comparisons, followed by Mann-Whitney (MW) *U* test with Bonferroni correction. Statistical analyses were conducted using SPSS for Windows (Release 14.0K, IBM SPSS Inc., USA).

## Results

**Fig. 1:**
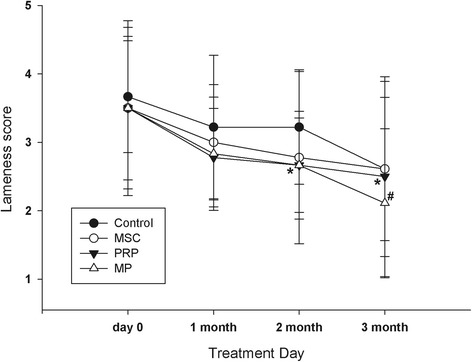
The lameness score of the OA dogs. Control, treated with 1 ml of PBS; PRP, treated with 1 ml of PRP; MSC, treated with 1.0 × 10^7^ MSC in 1 ml of PBS; MP, treated with 1.0 × 10^7^ MSC cell in 1 ml of PRP. *Asterisk* indicates significantly different with day 0 within PRP group by MW test; *Number sign* significantly different with day 0 within MP group by MW test

Although the lameness score in MP group was decreased compared to those in the other groups, there were no significant changes between groups. The lameness score was significantly decreased at 2 months and at 3 months after treatment in the PRP group and MP group when compared with the previous treatment, respectively (Fig. 1).

**Fig. 2:**
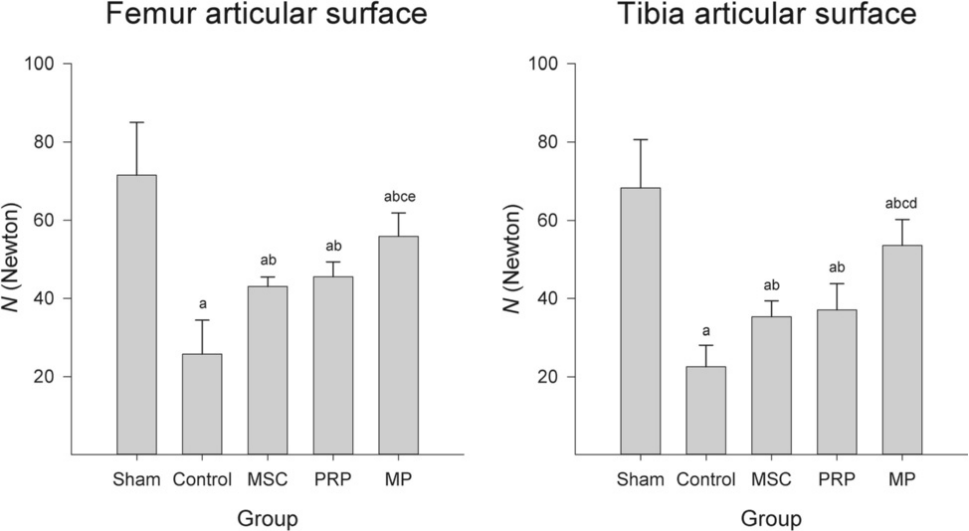
Focal compressive strengths on the femoral and tibial articular cartilages. Values are expressed as mean ± SD of six dogs, *N* (Newton). ^a^ *p* < 0.05 as compared with sham control by LSD test; ^b^ *p* < 0.05 as compared with control by LSD test; ^c^ *p* < 0.05 as compared with MSC treated group by LSD test; ^d^ *p* < 0.01 and ^e^ *p* < 0.05 as compared with PRP treated group by LSD test

The focal compressive strength of the femoral and tibial articular surface cartilages were significantly decreased in the control group as compared to that of the sham group. However, the focal compressive strength significantly increased by the treatment of all three test materials. In MP group, the focal compressive strength was higher than those in any other treated groups (Fig. 2).

The Mankin scores of control group were significantly increased as compared with those of shame group. When compared with control group, it was found that the Mankin score significantly reduced with the treatment of all three test materials on the femoral and tibial articular cartilages. Especially, the Mankin score of MP treated dogs was lowest compared to those of MSC or PRP treated dogs (Table 2).

**Table 2:** Mankin scores on the femur and tibia articular cartilages

Groups	Femur					Tibia				
	Surface damage	Hypocellularity	Clone	Stain intensity	Totalized (max = 12)	Surface damage	Hypocellularity	Clone	Stain intensity	Totalized (max = 12)
Sham	0.33 ± 0.52	0.17 ± 0.41	0.17 ± 0.41	0.33 ± 0.52	1.00 ± 0.63	0.17 ± 0.41	0.33 ± 0.52	0.17 ± 0.41	0.17 ± 0.41	0.83 ± 1.60
Control	2.50 ± 0.55^a^	1.67 ± 0.52^a^	1.33 ± 0.82^a^	1.67 ± 0.52^a^	7.17 ± 1.17^a^	2.17 ± 0.41^a^	2.17 ± 0.41^a^	1.00 ± 0.63^a^	1.50 ± 0.55^a^	6.83 ± 1.17^a^
MSC	1.00 ± 0.63^bc^	0.83 ± 0.41^bc^	1.17 ± 0.41^a^	1.17 ± 0.41^b^	4.17 ± 0.75^ac^	0.67 ± 0.52^c^	1.17 ± 0.41^bc^	0.67 ± 0.52	1.33 ± 0.52^a^	3.83 ± 0.75^ac^
PRP	0.83 ± 0.41^c^	0.83 ± 0.41^bc^	0.83 ± 0.41^b^	1.00 ± 0.00	3.50 ± 0.55^ac^	0.50 ± 0.55^c^	0.83 ± 0.75^c^	0.67 ± 0.52	1.17 ± 0.41^a^	3.17 ± 0.98^ac^
MP	0.50 ± 0.55^c^	0.33 ± 0.52^c^	0.33 ± 0.52^cf^	0.33 ± 0.52^cf^	1.50 ± 1.38^ceg^	0.33 ± 0.52^c^	0.33 ± 0.52^cf^	0.17 ± 0.41^c^	0.50 ± 0.55^ceh^	1.33 ± 1.21^ceh^

Values are expressed as mean ± SD of six dogs

*MSC* mesenchymal stem cells, *PRP* platelet-rich plasma, *MP* MSC and PRP co-treatment

^a^ *p* < 0.01 as compared with sham control by LSD test

^b^ *p* < 0.05 as compared with sham control by LSD test

^c^ *p* < 0.01 as compared with control by LSD test

^d^ *p* < 0.05 as compared with control by LSD test

^e^ *p* < 0.01 as compared with MSC treated group by LSD test

^f^ *p* < 0.05 as compared with MSC treated group by LSD test

^g^ *p* < 0.01 as compared with PRP treated group by LSD test

^h^ *p* < 0.05 as compared with PRP treated group by LSD test

**Fig. 3:**
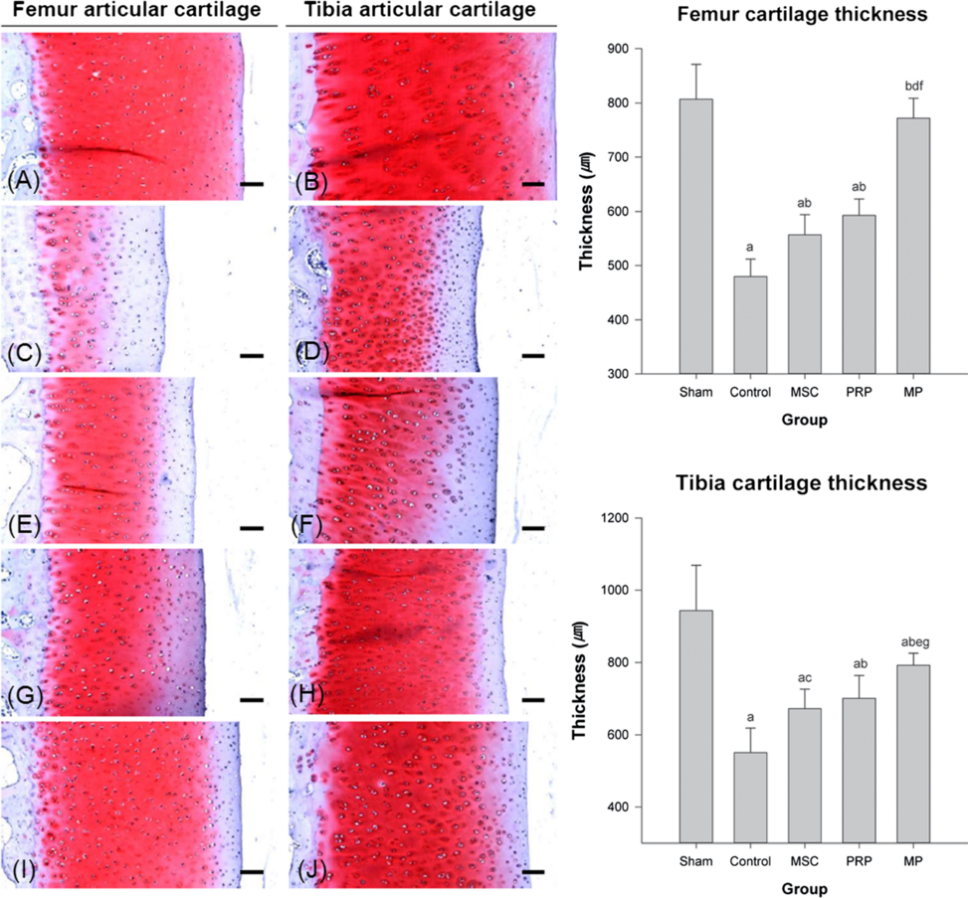
Representative general histopathological images and the thicknesses of the femoral and tibial articular cartilages. Of sham (**a**, **b**), control (**c**, **d**), MSC (**e**, **f**), PRP (**g**, **h**), and MP (**i**, **j**). H&E and Safranin O stain. *Scale bars* = 90 μm. ^a^ *p* < 0.01 as compared with sham control by LSD test; ^b^ *p* < 0.01 and ^c^ *p* < 0.05 as compared with control by LSD test; ^d^ *p* < 0.01 as compared with MSC treated group by LSD test; ^e^ *p* < 0.01 and ^f^ *p* < 0.05 as compared with PRP treated group by LSD test

The thickness of articular cartilages was higher in the MSC and PRP than control group. The more favorable effect on the articular surface was examined in MP group, compared to those of MSC and PRP groups (Fig. 3).

**Fig. 4:**
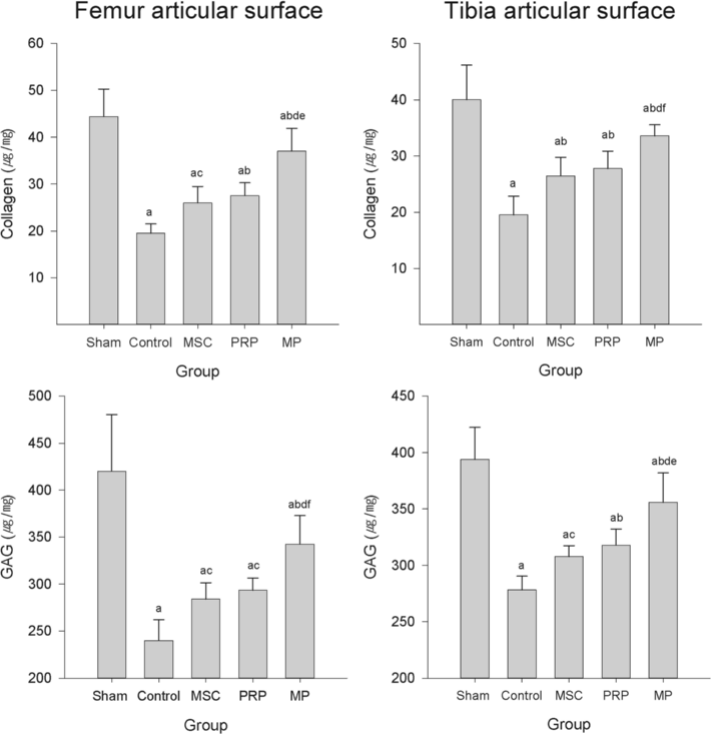
COL and GAG contents on the femoral articular cartilages. ^a^ *p* < 0.01 as compared with sham control by LSD test; ^b^ *p* < 0.01 and ^c^ *p* < 0.05 as compared with control by LSD test; ^d^ *p* < 0.01 as compared with MSC treated group by LSD test; ^e^ *p* < 0.01 and ^f^ *p* < 0.05 as compared with PRP treated group by LSD test

The contents of COL and GAG as the main component of ECM were significantly decreased in control group compared with those of sham group, but it was significantly increased in all treated groups compared with control group (Fig. 4).

**Fig. 5:**
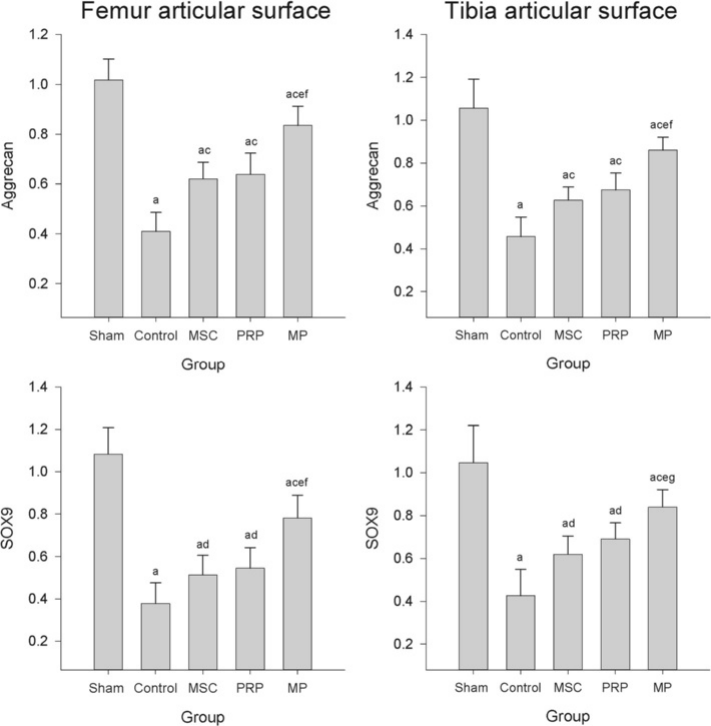
mRNA expressions of aggrecan and SOX9 on the femoral and tibial articular cartilages. Values are expressed as mean ± SD of six dogs, relative to control/G3PDH mRNA. ^a^ *p* < 0.01 and ^b^ *p* < 0.05 as compared with sham control by LSD test; ^c^ *p* < 0.01 and ^d^ *p* < 0.05 as compared with control by LSD test; ^e^ *p* < 0.01 as compared with MSC treated group by LSD test; ^f^ *p* < 0.01 and ^g^ *p* < 0.05 as compared with PRP treated group by LSD test

When the real-time PCR performed to determine the ECM-related genes, the expression of cartilage aggrecan and SOX9 was decreased as compared with those of sham group. However, those down-regulated gene expressions were significantly increased by the treatment of all three test materials, and it was most potent in MP group (Fig. 5).

**Fig. 6:**
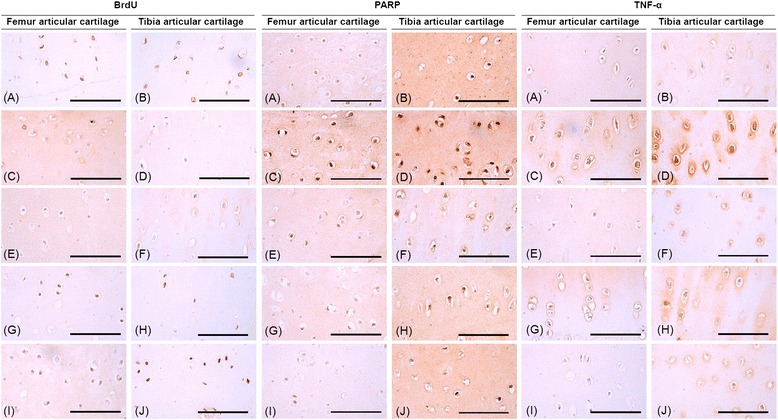
Representative immunohistochemical findings of femoral and tibial articular cartilage (BrdU, TNF-α, and PARP). Of sham (**a**, **b**), control (**c**, **d**), MSC (**e**, **f**), PRP (**g**, **h**), and MP (**i**, **j**). *Scale bars* = 90 μm

The BrdU-positive cells in the femoral and tibial articular cartilages significantly decreased in control group comparing to that of sham group. The values were significantly increased in all treated groups compared with control group. The increase of cell proliferation on the cartilage was most significant in MP group than MSC or PRP group (Fig. 6, Tables 3 and 4).

**Table 3:** Immunohistochemical analysis on the femur articular cartilages

Cell numbers (cells/mm^2^)	Sham	Control	MSC	PRP	MP
BrdU+	144.17 ± 47.83	9.00 ± 2.83^f^	55.00 ± 17.34^fg^	77.17 ± 20.36^g^	129.67 ± 31.39^fghi^
IL-1β+	14.67 ± 4.18	140.50 ± 16.69^a^	50.83 ± 15.77^ab^	44.50 ± 13.66^ab^	23.50 ± 7.87^bcd^
TNF-α+	5.50 ± 4.09	103.00 ± 18.70^f^	55.33 ± 18.91^fg^	38.00 ± 10.16^fg^	13.83 ± 3.66^ghi^
COX-2+	16.33 ± 2.73	102.67 ± 22.57^f^	65.50 ± 10.21^fg^	44.50 ± 10.21^fg^	23.67 ± 6.92^ghi^
iNOS+	57.83 ± 15.82	196.67 ± 42.57^a^	106.50 ± 17.60^ab^	66.17 ± 10.53^b^	40.33 ± 12.40^bce^
Caspase-3+	16.50 ± 8.87	80.33 ± 11.22^a^	52.17 ± 12.62^ab^	46.17 ± 11.86^ab^	18.00 ± 8.46^bcd^
PARP+	30.00 ± 11.92	101.17 ± 21.64^a^	64.17 ± 14.63^ab^	54.50 ± 13.16^ab^	32.50 ± 10.31^bce^
IFN-γ+	5.33 ± 3.27	88.33 ± 13.35^a^	47.17 ± 11.51^ab^	31.17 ± 15.28^ab^	12.50 ± 2.88^bcd^

Values are expressed as mean ± SD of six dogs

*MSC* mesenchymal stem cells, *PRP* platelet-rich plasma, *MP* MSC and PRP co-treatment

^a^ *p* < 0.01 as compared with sham control by LSD test

^b^ *p* < 0.01 as compared with control by LSD test

^c^ *p* < 0.01 as compared with MSC treated group by LSD test

^d^ *p* < 0.01 as compared with PRP treated group by LSD test

^e^ *p* < 0.05 as compared with PRP treated group by LSD test

^f^ *p* < 0.01 as compared with sham control by MW test

^g^ *p* < 0.01 as compared with control by MW test

^h^ *p* < 0.01 as compared with MSC treated group by MW test

^i^ *p* < 0.01 as compared with PRP treated group by MW test

**Table 4:** Immunohistochemical analysis on the tibia articular cartilages

Cell numbers (cells/mm^2^)	Sham	Control	MSC	PRP	MP
BrdU+	123.67 ± 24.40	7.00 ± 2.97^h^	49.50 ± 12.69^hj^	62.00 ± 16.09^hj^	120.33 ± 29.27^jkl^
IL-1β+	5.33 ± 2.16	170.67 ± 19.94^a^	63.50 ± 11.64^ac^	41.33 ± 16.10^ac^	15.67 ± 10.25^cdf^
TNF-α+	6.33 ± 3.14	141.00 ± 32.51^a^	70.33 ± 14.17^ac^	53.33 ± 15.24^ac^	28.50 ± 10.45^bcdg^
COX-2+	4.00 ± 2.61	129.67 ± 44.74^h^	61.67 ± 13.76^hj^	37.50 ± 11.04^hj^	18.33 ± 3.93^hjkl^
iNOS+	41.17 ± 17.88	243.00 ± 54.32^h^	124.33 ± 45.37^hj^	73.00 ± 10.97^ij^	47.60 ± 13.17^ikl^
Caspase-3+	6.00 ± 3.85	102.50 ± 27.06^h^	43.17 ± 12.48^hj^	25.83 ± 5.53^hj^	15.33 ± 4.63^hjk^
PARP+	45.67 ± 10.63	134.67 ± 38.53^a^	68.33 ± 17.49^c^	63.33 ± 19.03^c^	39.67 ± 12.36^ceg^
IFN-γ+	7.50 ± 2.43	49.67 ± 11.40^h^	28.33 ± 5.79^hj^	22.33 ± 3.27^hj^	13.83 ± 3.82^jkl^

Values are expressed as mean ± SD of six dogs

*MSC* mesenchymal stem cells, *PRP* platelet-rich plasma, *MP* MSC and PRP co-treatment

^a^ *p* < 0.01 as compared with sham control by LSD test

^b^ *p* < 0.05 as compared with sham control by LSD test

^c^ *p* < 0.01 as compared with control by LSD test

^d^ *p* < 0.01 as compared with MSC treated group by LSD test

^e^ *p* < 0.05 as compared with MSC treated group by LSD test

^f^ *p* < 0.01 as compared with PRP treated group by LSD test

^g^ *p* < 0.05 as compared with PRP treated group by LSD test

^h^ *p* < 0.01 as compared with sham control by MW test

^i^ *p* < 0.05 as compared with sham control by MW test

^j^ *p* < 0.01 as compared with control by MW test

^k^ *p* < 0.01 as compared with MSC treated group by MW test

^l^ *p* < 0.01 as compared with PRP treated group by MW test

Immunopositive cells to caspase-3 and PARP were significantly increased in the femoral and tibial cartilages of control group when compared with those of the sham group. However, these increased cells were significantly reduced by treatment of all three test materials, and the reduction was most significant in MP group (Fig. 6, Tables 3 and 4).

TNF-α, COX-2, IL-1β, iNOS, and IFN-γ staining cells were increased significantly in the femoral and tibial cartilages of control group compared with sham group. However, these increases of pro-inflammatory cytokines on the cartilages were diminished in treated groups (Fig. 6 and Additional file 1: Fig. S1-S5, Tables 3 and 4).

## Discussion

Osteoarthritis (OA) is characterized by the loss of articular cartilage components with inflammation, eventually resulting in impaired joint function [24, 25]. For this reason, this study mainly focused on the evaluation of the clinical signs, the change of ECM component and articular cartilage, the gene expression related to chondrogenesis, and the articular pathologic processes such as inflammation and apoptosis with the treatment of PRP and/or MSC.

Previously, it has been reported that the intra-articular injection of PRP reduced the lameness score [26]. Similarly, there were a meaningful decrease of lameness score with the treatment of PRP and MSC mixed with PRP in this canine OA model. Based on this result, we anticipate that PRP would relieve pain and improve articular function in an arthritic condition.

It is well known that the structure of articular cartilage is altered by the disorganization of collagen network, decrease of proteoglycan contents, and disruption of the integrity of the ECM in the process of OA [27]. Therefore, we tested the compressive strength of the articular cartilage to observe whether PRP or MSCs has a protective effect on the articular damage by OA. As previous studies have reported, Mankin score is a good index of osteoarthritis [28] and it is highly associated with compressive strength of articular cartilage [27]. We evaluated Mankin score of the affected articular cartilage. In addition, the thickness of articular cartilage and the content of COL and GAG were measured to confirm the OA condition. As a result, there were decreases in Mankin score as well as increases in the thickness of cartilage and the content of COL and GAG in the PRP and/or MSC treatment. These results suggested that PRP and MSC may have protective and regenerative effects on the degenerative cartilage of OA.

Aggrecan is known as a core protein of cartilage-specific proteoglycan, and it was reported that the mRNA expression of aggrecan was down-regulated according to the severity of OA condition [29, 30]. The expression of SOX9, the essential transcription factor for the chondrogenic differentiation, was also inhibited by OA model [30]. In this study, the down-regulations of SOX-9 and aggrecan in the control group were increased by the MSC and PRP treatment. Based on these results, we can suppose that the increase of ECM-related factors such as aggrecan, SOX9, COL, and GAG may be closely associated with the recovery of damaged articular cartilage. From this aspect, it could be explained by the fact that PRP and/or MSC treatment was associated with the increase of focal compressive strengths and the decrease of Mankin score. These findings are compatible with a previous report indicated that the inhibition of SOX9 gene expression and the content of GAG might result in cartilage degeneration [30].

We next performed BrdU staining to examine whether the MSC and/or PRP treatment promoted the proliferation of chondrocytes. It was shown that BrdU-positive cells decreased in OA condition and increased with a treatment of MSC and/or PRP. We also performed immunostaining for the caspase-3 and PARP expression examine whether apoptotic change during OA could be affected by PRP and/or MSC treatment. Results showed that the number of caspase-3- and PARP-positive cells was increased in the OA condition and was reduced with a treatment of MSC and/or PARP. These results were consistent with the findings of previous study that caspase-3 led to PARP to cleavage and consequently caused the progression of OA with cell apoptosis and death [31]. Therefore, we propose that the proliferation of chondrocyte may be suppressed in the process of OA through apoptotic change of articular cartilage, and this may be partly prevented by MSC and/or PRP treatment.

We examined the protein expression of cytokines relative to inflammatory reaction in the cartilage tissue. It has been known that pro-inflammatory cytokines mediated the activation of various inflammatory pathways and played a role in the progression of OA [32]. In the present study, the numbers of TNF-α-, COX-2-, IL-1β-, IFN-γ-, and iNOS-positive cells were increased in the OA condition, and MSC and/or PRP treatment stimulated the down-regulated expression of inflammatory cytokines. This result is comparable to the previous studies that MSC increased the anti-inflammatory cytokines such as IL-10, and PRP also had anti-inflammatory effect via blockage of NF-κB cascade, which contributes to the up-regulation of pro-inflammatory cytokines [33]. It has been known that MSC played important role in the cartilage repair by direct differentiation to chondrocyte and paracrine effect [8, 34]. In addition, it also has been reported that PRP could promote the proliferation by its various growth factors rather than differentiation of MSCs [35]. Therefore, PRP might prevent the depletion of MSCs, enhance the effect of MSCs, and guide MSCs to properly differentiate into chondrocytes. Consistent with previous study, the results of this study suggested that each treated with either MSC or PRP could decrease death or apoptosis of chondrocytes and inhibit inflammatory response [31, 36] and that the co-treatment with MSC and PRP might have beneficial effects on the pathologic changes in the articular cartilage.

In summary, we have shown that MSC and/or PRP ameliorated the degeneration of articular cartilage in the surgically induced OA animal model. The proliferation of chondrocyte may be suppressed in the process of OA through apoptotic change of articular cartilage, and this may be partly prevented by MSC and/or PRP treatment. MSC and/or PRP treatment also stimulated the down-regulated expression of inflammatory cytokines such as TNF-α-, COX-2-, IL-1β-, IFN-γ-, and iNOS. It will be needed to elucidate if transplanted MSCs are engrafted and differentiated to chondrocyte in OA cartilage and how MSCs and PRP interact in intra-articular environment.

## Conclusions

Taken together, this study shows that the combination of MSC and PRP has a beneficial and synergistic effect on OA via the ECM synthesis and chondrocyte proliferation and via the anti-inflammatory reaction. Therefore, the combination treatment of MSC and PRP may be very useful as an inflammatory regulator for the treatment of OA that exhibit irreversible articular degeneration.

### Ethics approval and consent to participate

All procedures were approved (KNU2015-060) by the Institutional Animal Care and Use Committees of Kyungpook National University.

## Additional file

Additional file 1:
**This contains Figures S1–S6 and Table S1. Fig. S1-S5) Representative images of IL-1β+,COX-2+, iNOS+, Caspase-3+ and IFN-γ+ cells, Fig. S6) Result of FACS analysis, Table S1) Primary antisera and detection kits for immunohistochemistry used in this study **

## Abbreviations

BrdU: 5-bromo-2′-deoxyuridine
CD: cluster of differentiation
COX: cyclooxygenase
ECM: extracellular matrix
GAG: glycosaminoglycan
IFN: interferon
IL: interleukin
iNOS: inducible nitric oxide synthase
LSD: least-significant differences
MSC: mesenchymal stem cell
MW: Mann-Whitney
OA: osteoarthritis
PARP: cleaved poly (ADP-ribose) polymerase
PBS: phosphate-buffered saline
PRP: platelet-rich plasma
TNF: tumor necrosis factor
