# Supplementary material for: Adipose-derived mesenchymal stem cells and platelet-rich plasma synergistically ameliorate the surgical-induced osteoarthritis in Beagle dogs
Source: J Orthop Surg Res. 2016 Jan 15;11:9. doi: 10.1186/s13018-016-0342-9 (PMC4714505; doi:10.1186/s13018-016-0342-9)
Supplement: Additional file 1: — This contains Figures S1–S6 and Table S1. Fig. S1-S5) Representative images of IL-1β+,COX-2+, iNOS+, Caspase-3+ and IFN-γ+ cells, Fig. S6) Result of FACS analysis, Table S1) Primary antisera and detection kits for immunohistochemistry used in this study [file 13018_2016_342_MOESM1_ESM.docx]

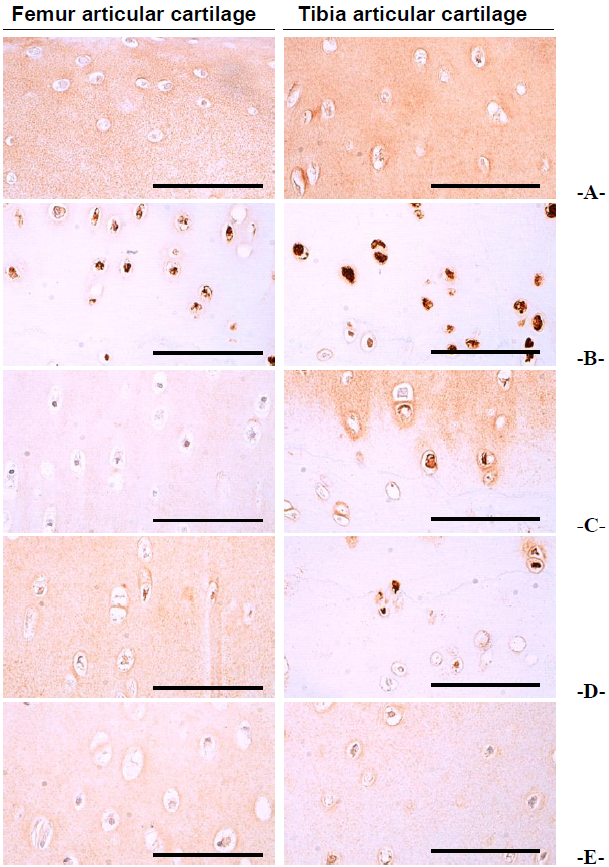


Figure S1. Representative images of IL-1β+ cells, taken from the femur and tibia articular surface cartilages of sham or OA dogs. **A** = Sham: Sham-operated vehicle control; **B** = OA control: Surgical OA-induced vehicle control; **C** = MSC: Surgical OA-induced and MSC treated group; **D** = PRP: Surgical OA-induced and PRP treated group; **E** = MP: Surgical OA-induced, and MSC and PRP co-treated group. Scale bars = 90 μm.


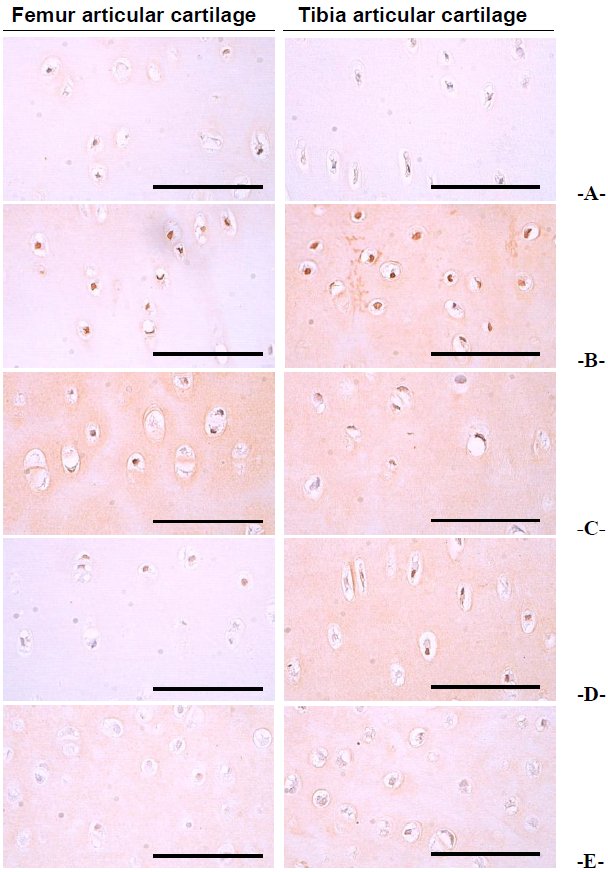


Figure S2. Representative images of COX-2+ cells, taken from the femur and tibia articular surface cartilages of sham or OA dogs. **A** = Sham: Sham-operated vehicle control; **B** = OA control: Surgical OA-induced vehicle control; **C** = MSC: Surgical OA-induced and MSC treated group; **D** = PRP: Surgical OA-induced and PRP treated group; **E** = MP: Surgical OA-induced, and MSC and PRP co-treated group. Scale bars = 90 μm.


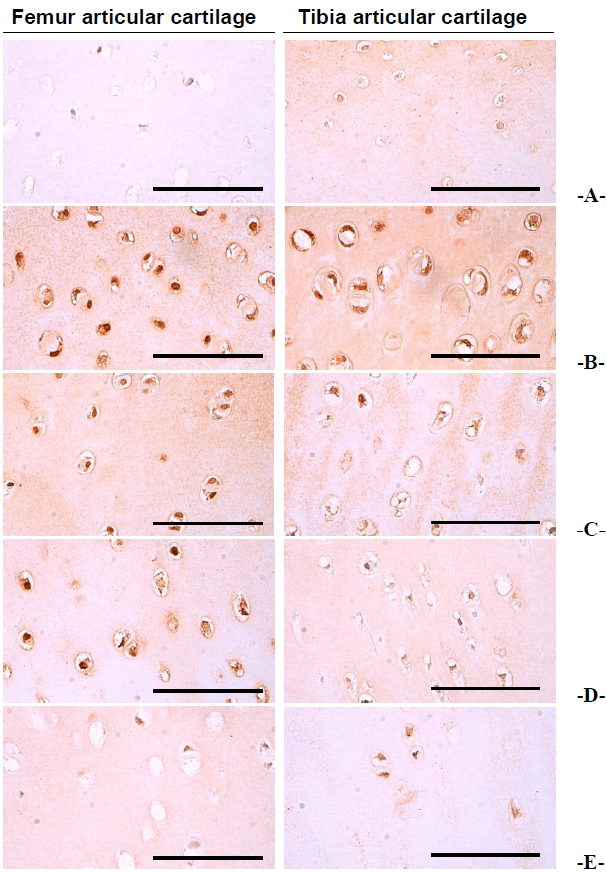


Figure S3. Representative images of iNOS+ cells, taken from the femur and tibia articular surface cartilages of sham or OA dogs. **A** = Sham: Sham-operated vehicle control; **B** = OA control: Surgical OA-induced vehicle control; **C** = MSC: Surgical OA-induced and MSC treated group; **D** = PRP: Surgical OA-induced and PRP treated group; **E** = MP: Surgical OA-induced, and MSC and PRP co-treated group. Scale bars = 90 μm.


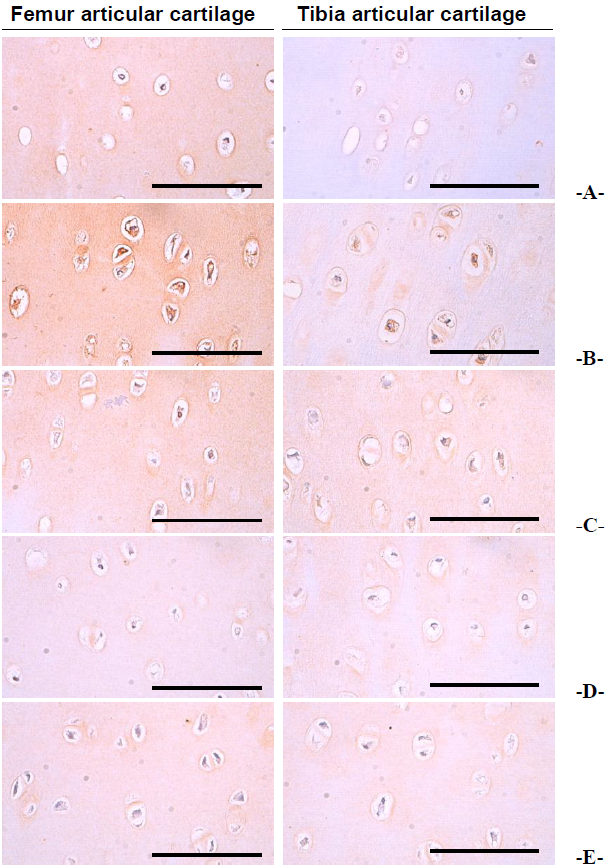


Figure S4. Representative images of Caspase-3+ cells, taken from the femur and tibia articular surface cartilages of sham or OA dogs. **A** = Sham: Sham-operated vehicle control; **B** = OA control: Surgical OA-induced vehicle control; **C** = MSC: Surgical OA-induced and MSC treated group; **D** = PRP: Surgical OA-induced and PRP treated group; **E** = MP: Surgical OA-induced, and MSC and PRP co-treated group. Scale bars = 90 μm.


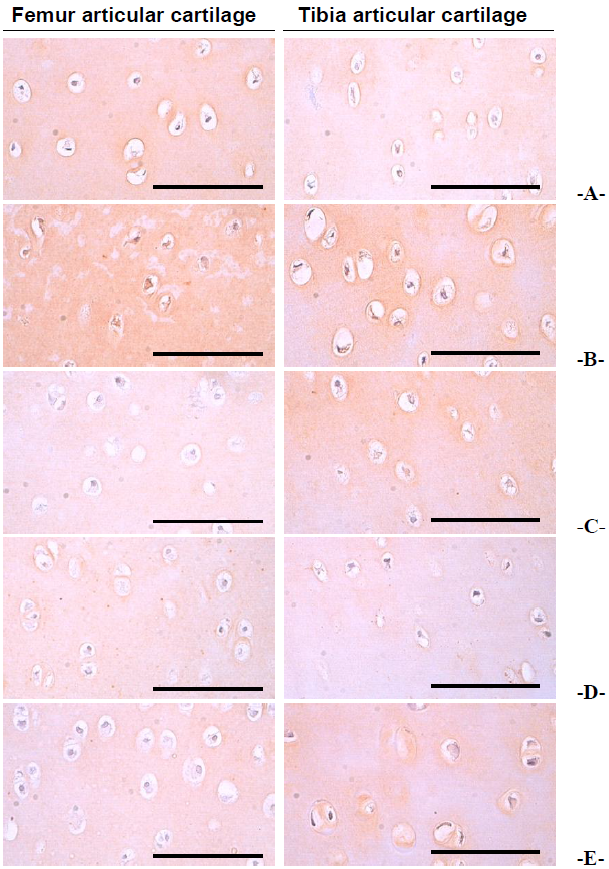


Figure S5. Representative images of IFN-γ+ cells, taken from the femur and tibia articular surface cartilages of sham or OA dogs. **A** = Sham: Sham-operated vehicle control; **B** = OA control: Surgical OA-induced vehicle control; **C** = MSC: Surgical OA-induced and MSC treated group; **D** = PRP: Surgical OA-induced and PRP treated group; **E** = MP: Surgical OA-induced, and MSC and PRP co-treated group. Scale bars = 90 μm.


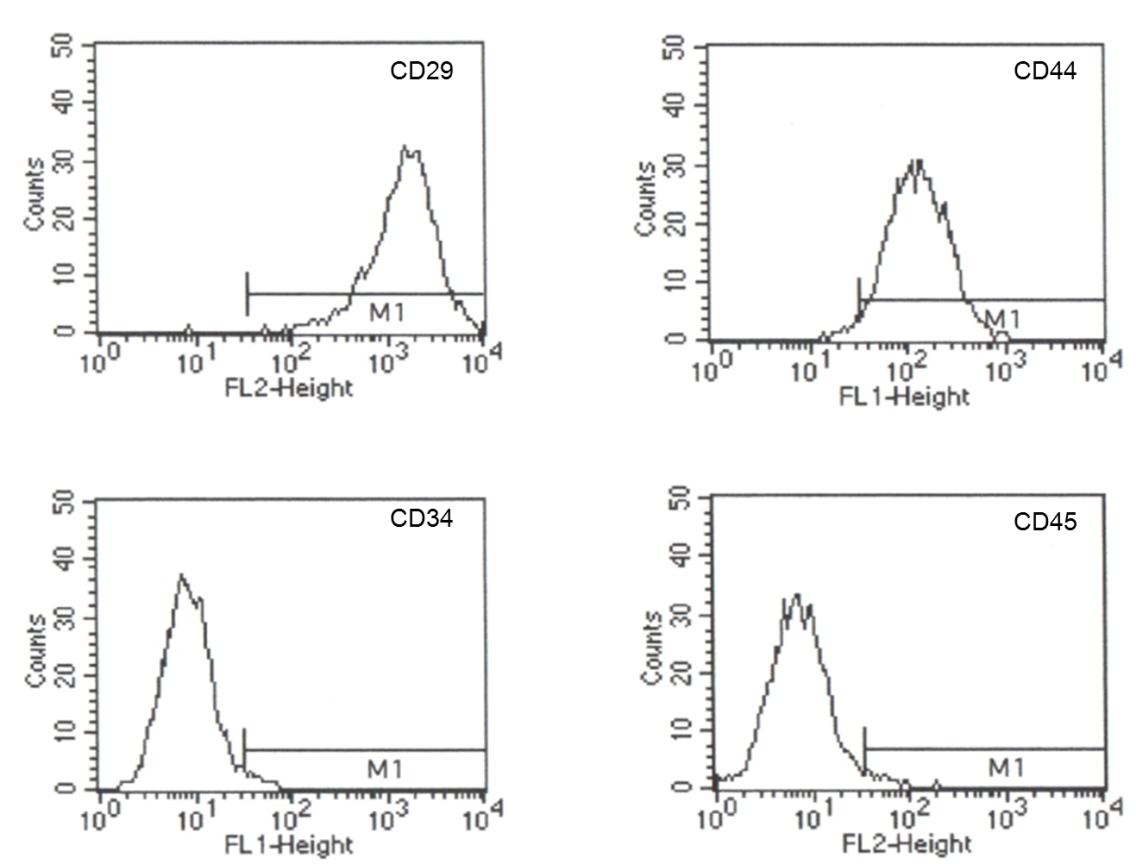


Figure S6. Result of FACS analysis. The obtained MSCs were positive for CD29 and CD44, and negative for CD34 and CD45.

Table S1**.** Primary antisera and detection kits for immunohistochemistry used in this study

| Antisera or detection kits | Code | Source | Dilution |
| --- | --- | --- | --- |
| Primary antisera* |  |  |  |
| Anti-5-Bromo-2’-Deoxyuridine antibody | RPN202 | Sigma-Aldrich, St. Louise, MO, USA | 1:200 |
| Anti-cleaved PARP (Asp214) specific antibody | 9545 | Cell Signaling Technology Inc, Danvers, MA, USA | 1:100 |
| Anti-cleaved caspase-3 (Asp175) polyclonal antibody | 9661 | Cell Signaling Technology Inc, Danvers, MA, USA | 1:200 |
| Anti-cyclooxygenase-2 (murine) polyclonal antibody | 160126 | Cayman Chemical., Ann Arbor, MI, USA | 1:200 |
| Anti-interferon γ antibody | AF781 | R&D System, Minneapolis, MN, USA | 1:100 |
| Anti-interleukin-1β (H-153) polyclonal antibody | sc-7884 | Santa Cruz Biotechnology, Santa Cruz, CA, USA | 1:100 |
| Anti-nitric oxide synthase2 (N-20) polyclonal antibody | sc-651 | Santa Cruz Biotechnology, Santa Cruz, CA, USA | 1:100 |
| Anti-tumor necrosis factor-α antibody | sc-52746 | Santa Cruz Biotechnology, Santa Cruz, CA, USA | 1:200 |
| Detection kits |  |  |  |
| Vectastain Elite ABC Kit | PK-6200 | Vector Lab. Inc., Burlingame, CA, USA | 1:50 |
| Peroxidae substrate Kit | SK-4100 | Vector Lab. Inc., Burlingame, CA, USA | 1:50 |

*All antisera were diluted using 0.01M phosphate buffered saline. PARP = Cleaved poly(ADP-ribose) polymerase
